# Inflammatory Mediators Drive Adverse Right Ventricular Remodeling and Dysfunction and Serve as Potential Biomarkers

**DOI:** 10.3389/fphys.2018.00609

**Published:** 2018-05-23

**Authors:** Akylbek Sydykov, Argen Mamazhakypov, Aleksandar Petrovic, Djuro Kosanovic, Akpay S. Sarybaev, Norbert Weissmann, Hossein A. Ghofrani, Ralph T. Schermuly

**Affiliations:** ^1^Excellence Cluster Cardio-Pulmonary System, Universities of Giessen and Marburg Lung Center, German Center for Lung Research, Justus Liebig University of Giessen, Giessen, Germany; ^2^Department of Mountain and Sleep Medicine and Pulmonary Hypertension, National Center of Cardiology and Internal Medicine, Bishkek, Kyrgyzstan

**Keywords:** right ventricle, adverse remodeling, dysfunction, failure, inflammation

## Abstract

Adverse right ventricular (RV) remodeling leads to ventricular dysfunction and failure that represents an important determinant of outcome in patients with pulmonary hypertension (PH). Recent evidence indicates that inflammatory activation contributes to the pathogenesis of adverse RV remodeling and dysfunction. It has been shown that accumulation of inflammatory cells such as macrophages and mast cells in the right ventricle is associated with maladaptive RV remodeling. In addition, inhibition of inflammation in animal models of RV failure ameliorated RV structural and functional impairment. Furthermore, a number of circulating inflammatory mediators have been demonstrated to be associated with RV performance. This work reviews the role of inflammation in RV remodeling and dysfunction and discusses anti-inflammatory strategies that may attenuate adverse structural alterations while promoting improvement of RV function.

## Introduction

Pulmonary arterial hypertension (PAH) is a severe and debilitating disease characterized by progressive remodeling of small pulmonary arteries leading to sustained pressure elevation in the pulmonary circulation. The resulting chronic pressure overload induces remodeling of the right ventricle. Although increased pulmonary pressure is caused by changes in the pulmonary vasculature, severity of symptoms and survival of patients with PAH are strongly dependent on the ability of the right ventricle to cope with chronically increased afterload (Chin et al., 2005).

Cardiac remodeling in general is defined as “genome expression, molecular, cellular, and interstitial changes that are manifested clinically as changes in size, shape and function of the heart after cardiac injury” (Cohn et al., 2000). Cardiac remodeling is a complex process that affects every cell type comprising the heart tissue and involves cardiomyocyte hypertrophy, fibrosis, metabolic changes, and angiogenesis. Right ventricular (RV) remodeling in response to pressure overload represents a number of adaptations in the size, shape, structure, and function of the right ventricle. Hypertrophy of the RV wall allows compensating for increased afterload and helps maintain cardiac output in PAH patients. However, geometric adaptation of the right ventricle in PAH is heterogeneous. Although PAH is associated with a spectrum of RV geometric adaptation, two patterns of RV hypertrophy can be distinguished based on the RV mass to volume (M/V) ratio: eccentric hypertrophy with low M/V ratio and concentric hypertrophy with high M/V ratio (Badagliacca et al., 2015). Concentric hypertrophy represents a more favorable RV adaptive remodeling pattern in patients with PAH and is associated with minimal fibrosis and preserved RV function and cardiac output (Vonk-Noordegraaf et al., 2013). In contrast, eccentric hypertrophy represents maladaptive RV remodeling and is associated with excessive RV fibrosis, RV dysfunction, and decreased cardiac output (Vonk-Noordegraaf et al., 2013). However, the mechanisms underlying adverse RV remodeling and dysfunction are poorly understood. A better understanding of the mechanisms of the RV remodeling may help identify candidate targets for novel therapeutic strategies directed specifically at the right ventricle and thus improve survival in these patients.

There is substantial evidence for the important role of inflammation in the pathogenesis of PAH (Hassoun et al., 2009; El Chami and Hassoun, 2011; Price et al., 2012). Furthermore, elevated levels of circulating mediators of inflammation correlate with disease severity, symptom burden and survival in PAH patients (Humbert et al., 1995; Quarck et al., 2009; Soon et al., 2010; Cracowski et al., 2014; Heresi et al., 2014; Matura et al., 2015; Al-Naamani et al., 2016). Moreover, circulating inflammatory mediators released from the pulmonary vasculature might trigger or contribute to inflammatory processes in the right ventricle and thus adversely affect RV remodeling and function. Consistent with this view, chronic RV pressure overload without concomitant pulmonary vascular disease in patients with pulmonary stenosis is often associated with adaptive RV remodeling and preserved RV function, which remains compensated for decades (Haddad et al., 2008; Jurcut et al., 2011; Driessen et al., 2016, 2017). Another line of evidence suggesting the role of inflammation as a driver of adverse RV remodeling and dysfunction comes from studies of patients with systemic sclerosis or scleroderma-associated (SSc) PAH. Scleroderma is a systemic connective tissue disease characterized by chronic inflammation, fibrosis, and immune abnormalities (Furue et al., 2017). Although various mechanisms may underlie pulmonary hypertension (PH) in scleroderma patients, PAH due to vasculopathy of the small pulmonary arteries secondary to inflammation and fibrosis is one of the most frequent forms of PH in these patients (Launay et al., 2017). Patients with SSc-PAH have worse survival than patients with idiopathic PAH (Kato and Atsumi, 2017). Further, RV function strongly predicts survival in SSc-PAH patients (Mathai et al., 2011). Moreover, SSc-PAH patients have more impaired RV function compared with idiopathic PAH patients despite comparable afterload (Tedford et al., 2013; Mukherjee et al., 2017). Remarkably, significantly more inflammatory cells were found in RV tissue samples from SSc-PAH patients as compared to those from patients with idiopathic PAH thus confirming increased local inflammation in these patients (Overbeek et al., 2010). Contribution of inflammatory pathways to the pathogenesis of RV adverse remodeling and dysfunction is further supported by the association of circulatory mediators of inflammation with RV function in patients with PAH (Sztrymf et al., 2010; Yang et al., 2014; Prins et al., 2017). Interestingly, a recent magnetic resonance imaging investigation in individuals free of clinical cardiovascular disease has demonstrated that plasma levels of pro-inflammatory C-reactive protein (CRP) and interleukin-6 (IL-6) are independently associated with RV morphology, suggesting that systemic inflammation may contribute to RV structural changes (Harhay et al., 2013).

## Acute Right Ventricular Pressure Overload

Acute pulmonary embolism (PE) can produce a rapid and excessive elevation in afterload leading to acute RV dilatation, dysfunction and failure (Vitarelli et al., 2014; Grignola and Domingo, 2017). Importantly, RV dilatation and dysfunction in patients with acute PE are strong predictors of adverse clinical outcome (Coutance et al., 2011; Trujillo-Santos et al., 2013; Cho et al., 2014; Meinel et al., 2015). Interestingly, examination of autopsy RV tissue samples from patients who died from acute PE showed increased accumulation of neutrophilic granulocytes, lymphocytes, and macrophages in the RV wall suggesting that acute pressure overload-induced inflammatory response might contribute to RV failure after PE (Iwadate et al., 2001, 2003; Begieneman et al., 2008).

Experimental models of acute RV pressure overload induced by PE or pulmonary artery banding (PAB) confirmed that inflammation is one of the earliest events following pressure overload. In a rat model of acute PE induced by infusion of microspheres, RV failure after PE was associated with tissue infiltration of neutrophils and monocyte/macrophages (Watts et al., 2006, 2009). Neutrophil and macrophage infiltration in the right ventricle has also been demonstrated in a different model of acute RV failure induced by transient PAB in dogs (Dewachter et al., 2015). Increased mRNA expression of various chemokines (CCL-2, -3, -4, -6, -7, -9, -17, -20, -27; CXCL-1, -2, -3, -9, -10, -16; receptors CCR1 and CXCR4 in the right ventricle following acute elevation of afterload has been demonstrated (Watts et al., 2006, 2009; Zagorski et al., 2007, 2008; Dewachter et al., 2015). Notably, alterations in gene expression were more profound with increasing PH (Zagorski et al., 2008). Chemokines play important chemotactic roles for the recruitment of leukocytes to the site of inflammation and thus might promote leukocyte recruitment into the right ventricle during acute pressure overload. In addition, experimental acute RV failure was also associated with increased local expression of pro-inflammatory cytokines [IL-1β, IL-6, tumor necrosis factor α (TNF-α)] (Dewachter et al., 2010, 2015). Moreover, there was an inverse correlation between the right ventricle–pulmonary artery coupling and RV gene and protein expressions of IL-6 as well as to the RV neutrophil and macrophage infiltration (Dewachter et al., 2010).

## Chronic Right Ventricular Remodeling and Failure

Accumulating evidence from various experimental models of chronic RV pressure overload shows association of RV remodeling and dysfunction with increased inflammatory mediators in the right ventricle (Campian et al., 2010; Waehre et al., 2012; Belhaj et al., 2013; Guihaire et al., 2017; Luitel et al., 2017). The inflammatory mediators include pro-inflammatory cytokines, chemokines, as well as molecules secreted/released by inflammatory cells. The findings of studies investigating inflammatory markers in the RV tissue are summarized in **Table 1**.

**Table 1:** Summary of studies investigating inflammatory mediators in the right ventricular tissue.

Disease model	Species/subjects	Findings	References
Acute RVF following transient PAB	Dogs	Increased expression of CCL2, CCR2, IL-1β, IL-6, TNF-α mRNA.	Dewachter et al., 2010, 2015
Acute RVF following PE	Rats	Increased expression of the CC-chemokines (CCL-2, -3, -4, -6, -7, -9, -17, -20, -27), the CXC-chemokines (CXCL-1, -2, -3, -9, -10, -16), the receptors CCR1 and CXCR4, ICAM-1, selectin E, the cytokines IL-1β and IL-6 mRNA. Elevation of CCL2 protein expression. Increased MPO activity. Accumulation of neutrophils and monocyte/macrophages (CD68).	Watts et al., 2006, 2009; Zagorski et al., 2007, 2008
Acute RVF following PE	Autopsy tissues from patients	Increased recruitment of macrophages (CD68).	Iwadate et al., 2003
Acute RVF following transplantation	Human donors	Eight of 26 recipients (30.8%) developed RVF. Seven of these eight (87.5%) expressed TNF-α, but only 4 of the 18 (22.2%) who did not develop RVF expressed TNF-α. Higher TNF-α protein expression in the myocardium of donor hearts that developed RVF.	Birks et al., 2000
Chronic hypoxic PH	Mice	Increased accumulation of CD68 positive cells.	Nergui et al., 2014
MCT-induced PH	Rats	Increased TNF-α, IL-1, IL-6 mRNA expression. Elevated protein expression of TNF-α, NF-κB subunits p100/p52, and Rel-B. Increased accumulation of CD45^+^ cells enhanced MPO activity.	Handoko et al., 2009; Campian et al., 2010; de Man et al., 2012; Ahmed et al., 2014; Moreira-Goncalves et al., 2015; Paulin et al., 2015; Nogueira-Ferreira et al., 2016; Rice et al., 2016; Wang et al., 2016; Alencar et al., 2017; Brown et al., 2017
Sugen-injection induced PH	Athymic rats	Macrophage infiltration.	Guihaire et al., 2017
PH due to blockade of VEGF receptor and exposure to chronic hypoxia	Female ovariectomized rats	Increased IL-6 mRNA expression.	Frump et al., 2015
PH due to prolonged systemic-to-pulmonary shunting	Growing piglets	Increased TNF-α, IL-1α, IL-1b, and ICAM2 mRNA expression.	Rondelet et al., 2012; Belhaj et al., 2013
Chronic RVF remodeling following PAB	Mice	Increased mRNA expression of CCL-2, CCL-5, CX3CL-1, CXCL-6, CXCL-10, CXCL-16, CD45R, CD3, CD4, CD8, IL-6, TNF-α, Fn14, mMCP-2, 4, -5, -6, and CPA3. Increased density and activity of mast cells.	Vistnes et al., 2010; Waehre et al., 2012; Luitel et al., 2017
Chronic RVF following PAB	Rats	Increased expression of activated p65 (NF-κB). Increased density and activity of mast cells, enhanced accumulation of CD68-positive macrophages.	Olivetti et al., 1989; Yoshida et al., 2016
PAH	Autopsy tissues from patients	Increased tissue content of CD68 positive macrophages.	Nergui et al., 2014
SSc-PAH, IPAH and controls	Autopsy tissues from patients	RV’s from SSc-PAH patients showed significantly more inflammatory cells than those from IPAH and then of controls.	Overbeek et al., 2010

*PE, pulmonary embolism; PAB, pulmonary artery banding; MCT, monocrotaline; PH, pulmonary hypertension; PAH, pulmonary arterial hypertension; SSc-PAH, pulmonary arterial hypertension due to systemic sclerosis; IPAH, idiopathic pulmonary arterial hypertension; RV, right ventricle; RVF, right ventricular failure; ICAM2, intercellular adhesion molecule 2; CXCL, chemokine (C-X-C motif) ligand; CCL, CC-chemokine; CX3CL, chemokine (C-X3-C motif) ligand; CCR2, chemokine oxC-C receptor 2; IL, interleukin; TNF-α, tumor necrosis factor α; NF-κB, nuclear factor “kappa-light-chain-enhancer” of activated oxB-cells; mMCP, mouse mast cell protease; CPA3, mast cell carboxypeptidase A3; MPO, myeloperoxidase; Fn14, fibroblast growth factor-inducible molecule 14; VEGF, vascular endothelial growth factor*.

## Inflammatory Cells

Numerous studies have demonstrated that recruitment of immune cells from the circulatory system is important for the induction and maintenance of inflammatory processes in the heart (Sanchez-Trujillo et al., 2017). Although the precise role of inflammatory cells in the pathophysiology of maladaptive RV remodeling and dysfunction is not well established, accumulating evidence suggests that these cells might represent a potentially important target for management of RV adverse remodeling and failure.

### Macrophages

Resident macrophages are present in all tissues including the heart (Mylonas et al., 2015; Pinto et al., 2016). Expansion of cardiac macrophages has been documented in various experimental models of pressure overload-induced left ventricular remodeling and failure (Xia et al., 2009; Velten et al., 2012; Weisheit et al., 2014).

In contrast, data regarding the role of macrophages in RV remodeling and failure are still very limited. Similar to the left ventricular pressure overload, recruitment of macrophages into the right ventricle has been shown to be triggered by increased RV afterload induced by PAB in rats (Yoshida et al., 2016). Moreover, in a model of severe PH caused by a single injection of vascular endothelial growth factor (VEGF) receptors blocker in athymic rats, macrophage infiltration was associated with adverse RV remodeling and dysfunction (Guihaire et al., 2017). Increased macrophage recruitment has also been demonstrated in RV autopsy samples from PAH patients with RV failure (Nergui et al., 2014). Notably, autopsy samples from patients with SSc-associated PAH exhibited significantly more macrophage infiltration in the right ventricle as compared with those from patients with idiopathic PAH (Overbeek et al., 2010). The role of macrophage recruitment in the pathogenesis of pressure overload-induced RV remodeling has not yet been well studied. However, based on studies in left ventricular remodeling and failure, the potential mechanisms through which macrophages might contribute to RV remodeling and dysfunction include production of reactive oxygen species, regulation of cardiac inflammation and mediation of extracellular matrix alterations (Glezeva et al., 2015).

### Mast Cells

Mast cells are immune cells of the myeloid lineage and are residing in various tissues throughout the body including the heart (Dvorak, 1986; Rakusan et al., 1990). Mast cells play an important role in many inflammatory settings. Moreover, a growing body of evidence implicates mast cells in various cardiovascular diseases including left ventricular remodeling and failure (Levick et al., 2011). Enhanced accumulation of mast cells in hypertrophied and failing hearts suggests that mast cells play a role in the pathogenesis of these diseases (Panizo et al., 1995; Shiota et al., 2003; Batlle et al., 2006). Studies utilizing mast cell stabilizers, inhibitors of mast cell proteases, and mast cell deficient mice provided further evidence of the importance of mast cells for left ventricular remodeling and failure (Hara et al., 2002; Matsumoto et al., 2003; Levick et al., 2009; Li et al., 2016).

An early accumulation of mast cells in both left and right ventricles has been documented in a rat model of biventricular volume overload (Forman et al., 2006). Interestingly, the time course of the responses in the density of myocardial mast cells in this model was similar for the left and right ventricles (Brower et al., 2002). However, the number of mast cells after an initial increase returned to normal values by day 14 post-fistula. In rats, long-term pressure overload induced by PAB was associated with an increased mast cell density in the right ventricle (Olivetti et al., 1989). Similarly, in mice subjected to PAB, there was a time-dependent accumulation and activation of mast cells in the right ventricle (Luitel et al., 2017). In contrast, significant RV hypertrophy in rats born at simulated high altitude was not associated with an increase in cardiac mast cells density (Rakusan et al., 1990). Adaptive nature of RV remodeling in hypoxic PH may account for the unaltered mast cells density in the right ventricle in rat exposed to chronic hypoxia.

Mast cells granules contain proteases tryptase and chymase (Gilfillan and Beaven, 2011). In addition, mast cells generate a wide variety of cytokines, including IL-3, IL-4, IL-5, IL-6, IL-10, IL-13, IL-33, and TNFα; and chemokines, including CCL2, CCL3, CCL5, and CXCL8 (Gilfillan and Beaven, 2011). Mast cell mediators have been implicated in the stimulation of collagen synthesis leading to myocardial fibrosis or activation of matrix metalloproteinases causing collagen degradation and resulting in left ventricular dilatation (Levick et al., 2011). However, the role of mast cells in the pathogenesis of pressure overload-induced RV remodeling and dysfunction has not yet been studied in detail. Interestingly, elevated mRNA levels of mast cell proteases (Mcp)-2, 4, 5, 6, and carboxypeptidase A3 in the right ventricle have been observed in mice subjected to PAB suggesting their potential contribution to RV remodeling process (Luitel et al., 2017).

### Leukocytes

Numerous studies have shown that leukocytes play critical roles in the pathogenesis of left ventricular remodeling and failure in experimental models of left ventricular pressure overload (Damilano et al., 2011; Laroumanie et al., 2014; Nevers et al., 2015; Salvador et al., 2016). However, data on the leukocyte involvement in the RV remodeling and failure are limited. Several reports have demonstrated increased accumulation of CD45^+^ cells in the right ventricle of rats with monocrotaline-induced PH (Handoko et al., 2009; de Man et al., 2012; Brown et al., 2017). Notably, no leukocyte infiltration in the right ventricle was detected in compensated RV remodeling in rats with mild PH induced by injection of low doses of monocrotaline (Handoko et al., 2009; Brown et al., 2017). In mice subjected to PAB, pressure overload was associated with increased mRNA expression of leukocyte cell-surface gene markers, including CD45R, CD3, CD4, and CD8, in the right ventricle (Waehre et al., 2012).

## Inflammatory Mediators: Chemokines and Cytokines

The inflammatory mediators involved in ventricular remodeling and dysfunction can be produced by cells in the heart tissue, by infiltrating immune cells, or can be of extra-cardiac origin (Van Linthout and Tschope, 2017). Multiple factors may be responsible for the induction of inflammatory genes in the pressure-overloaded heart, including neurohumoral mediators, reactive oxygen species, as well as direct activation of mechanosensitive pro-inflammatory signals in the myocardium (Van Linthout and Tschope, 2017). Chemokines can recruit inflammatory cells into the pressure-overloaded myocardium. In addition to their chemotactic role, chemokines can stimulate release of pro-inflammatory cytokines, thus contributing to the amplified activation of inflammatory processes. Pro-inflammatory cytokines and chemokines can contribute to ventricular remodeling and dysfunction through various mechanisms, including negative inotropic effects on the myocardium, cardiomyocyte hypertrophy, cardiomyocyte apoptosis, and extracellular matrix alterations (Mann, 2015).

### Chemokines

Elevated plasma levels of the chemokines CXCL10, CXCL12, CXCL13, and CXCL16 have been observed in PAH patients (Heresi et al., 2010). Importantly, CXCL10, CXCL12, and CXCL16 levels significantly correlated with RV function (Yang et al., 2014). Elevated plasma levels of a chemokine CXCL9 have been observed in a murine model of PAB-induced RV pressure overload (Vistnes et al., 2010). Further, marked upregulation of several chemokines, including CCL2, CCL5, CXCL6, CXCL10, CXCL16, and CX3CL1, was detected in the right ventricle of mice subjected to PAB (Waehre et al., 2012). Similarly, in female ovariectomized rats with severe angioproliferative PH due to blockade of VEGF receptor and exposure to chronic hypoxia, upregulation of CCL2 mRNA in the right ventricle has been reported (Frump et al., 2015).

### TNF-α

TNF-α is a key multifunctional cytokine with pleiotropic actions in various inflammatory processes (Feldman et al., 2000). Although the normal heart does not express TNF-α, its mRNA and protein expression is rapidly induced in response to pressure overload (Kapadia et al., 1997). High levels of TNF-α have been shown to lead to left ventricular remodeling and dysfunction in animal models with long-term infusion of TNF-α, as well as in transgenic animals with targeted cardiac overexpression (Kubota et al., 1997; Bozkurt et al., 1998; Bryant et al., 1998). In contrast, TNF-α deficiency was associated with attenuation of cardiac inflammation and amelioration of adverse left ventricular remodeling and dysfunction in mice subjected to transverse aortic constriction (Sun et al., 2007). Consistent with findings from animal studies implicating TNF-α in the pathogenesis of heart failure, circulating levels of TNF-α have consistently been shown to be elevated in patients with chronic heart failure (Liu et al., 2014).

Involvement of TNF-α in the pathogenesis of RV failure has been suggested by demonstrating a relationship between TNF-α mRNA and protein expression in right ventricles of donor hearts immediately before implantation and the development of right heart failure early after transplantation (Birks et al., 2000). Further, a relationship between circulating levels of TNF-α and several parameters of RV failure, including severity of peripheral edema, RV ejection fraction and NYHA functional class, has been shown in patients with right heart failure related to ischemic heart disease or idiopathic dilated cardiomyopathy (Odeh et al., 2006). In line with these observations, expression of TNF-α has been shown to be upregulated in the right ventricle in various animal models of pressure overload-induced RV remodeling and failure, including PAB in mice (Luitel et al., 2017), monocrotaline-induced PH in rats (Ahmed et al., 2014; Moreira-Goncalves et al., 2015; Nogueira-Ferreira et al., 2016; Rice et al., 2016; Wang et al., 2016; Alencar et al., 2017), and prolonged overcirculation-induced PH in piglets (Rondelet et al., 2012; Belhaj et al., 2013). Notably, decompensated RV failure in monocrotaline-injected rats was associated with higher serum and RV myocardial TNF-α expression levels compared with compensated RV hypertrophy (Campian et al., 2010; Paulin et al., 2015).

The TNF superfamily comprises several members, including TNF-like weak inducer of apoptosis and its receptor fibroblast growth factor-inducible molecule 14 (Fn14), which have been implicated in pathological ventricular remodeling and heart failure (Novoyatleva et al., 2014). Recently, increased mRNA and protein expression of Fn14 in the pressure-overloaded right ventricles has been demonstrated (Novoyatleva et al., 2013). Moreover, mice deficient for Fn14 developed substantially less RV fibrosis and dysfunction following PAB compared to wild-type mice (Novoyatleva et al., 2013).

### IL-1

IL-1 cytokines consist of 11 members, including pro-inflammatory cytokines IL-1α and IL-1β that induce synthesis and expression of several hundreds of secondary inflammatory mediators (Dinarello, 2018). Upregulation of IL-1 has been consistently demonstrated in heart failure and is associated with worse prognosis (Van Tassell et al., 2015). Increased levels of IL-1β have also been shown in PAH patients (Humbert et al., 1995; Soon et al., 2010; Duncan et al., 2012; Cracowski et al., 2014; McMahan et al., 2015). Similarly, RV pressure overload in mice was associated with elevated plasma levels of IL-1α (Vistnes et al., 2010). Further, expression of IL-1α and IL-1b was upregulated in right ventricles of piglets subjected to prolonged systemic-to-pulmonary shunting (Rondelet et al., 2012; Belhaj et al., 2013) and rats with monocrotaline-induced PH (Rice et al., 2016) suggesting potential contribution of IL-1 cytokines to RV remodeling and dysfunction.

### IL-6

IL-6 is a multi-functional cytokine with a variety of biological activities, which has been implicated in left ventricular remodeling and failure (Fontes et al., 2015). Chronic IL-6 infusion in rats has been shown to lead to left ventricular dilatation and dysfunction (Janssen et al., 2005). In contrast, genetic deletion of IL-6 ameliorated pressure overload-induced adverse left ventricular remodeling and dysfunction in mice (Zhao et al., 2016). In patients with chronic heart failure, elevated circulating levels of IL-6 have been reported (Liu et al., 2014).

Numerous studies have documented increased levels of IL-6 in PAH patients (Humbert et al., 1995; Selimovic et al., 2009; Soon et al., 2010; Duncan et al., 2012; Cracowski et al., 2014; Heresi et al., 2014; Matura et al., 2015; Al-Naamani et al., 2016; Prins et al., 2017). Notably, increased expression of IL-6 mRNA has been detected in right ventricles of failing hearts (Plenz et al., 2001). In addition, IL-6 mRNA expression in the right ventricles inversely correlated with cardiac index in patients with advanced heart failure (Plenz et al., 2001). Similar findings were made in experimental models of RV pressure overload. Thus, elevated plasma levels of IL-6 and increased IL-6 expression in right ventricles has been demonstrated in rats with monocrotaline-induced PH (Wang et al., 2016), in mice subjected to PAB (Vistnes et al., 2010; Luitel et al., 2017), and in female ovariectomized rats with severe angioproliferative PH due to blockade of VEGF receptor and exposure to chronic hypoxia (Frump et al., 2015). Interestingly, in a recent study, a strong, independent, inverse relationship between IL-6 and RV morphology was demonstrated in asymptomatic individuals without documented cardiovascular disease (Harhay et al., 2013).

### Nuclear Factor-κB

The nuclear factor-κB (NF-κB) super family of transcription factors has been implicated in the regulation of a variety of physiological processes and plays a key role in regulation of inflammatory responses (Begalli et al., 2017). However, preclinical studies provided conflicting evidence for the role of NF-κB in left ventricular remodeling and failure. Several studies have demonstrated a critical role of NF-κB in promoting adverse cardiac remodeling and failure (Gupta et al., 2008; Maier et al., 2012), whereas other studies have suggested its importance in the development of compensatory hypertrophy following pressure overload (Andersen et al., 2012; Javan et al., 2015).

There have been only a few studies investigating the role of NF-κB in RV remodeling and dysfunction. In rats subjected to PAB, elevated expression of activated NF-κB has been found in right ventricles (Yoshida et al., 2016). Further, activation of the non-canonical NF-κB pathway with upregulation NF-κB subunits p100/p52 and Rel-B in right ventricles has been reported in rats with monocrotaline-induced PH (Nogueira-Ferreira et al., 2016). Interestingly, treatment of PAB rats with the NF-κB inhibitor pyrrolidine dithiocarbamate ameliorated RV inflammation and fibrosis and improved RV function (Yoshida et al., 2015).

## Inflammatory Mediators as Biomarkers

Circulating biomarkers serve as a non-invasive tool for diagnosis, assessment of the severity of the disease, prognosis, and response to treatment (Foris et al., 2013). A variety of inflammatory mediators, including IL-1α, IL-1b, IL-2, IL-4, IL-6, IL-8, IL-10, IL-13, IL-12p70, TNF-α, and CRP are upregulated in PAH (Quarck et al., 2009; Selimovic et al., 2009; Sztrymf et al., 2010; Duncan et al., 2012; Heresi et al., 2014; Matura et al., 2015; Al-Naamani et al., 2016; Prins et al., 2017). Importantly, markers of inflammation are associated with disease severity and mortality in these patients (Humbert et al., 1995; Soon et al., 2010; Cracowski et al., 2014; Karakurt et al., 2014; McMahan et al., 2015). However, there have been only a few studies that specifically addressed the relationship between circulating markers of inflammation and parameters of RV performance in patients with PAH. In patients with idiopathic PAH, elevated plasma levels of chemokines CXCL10, CXCL12, and CXCL16 correlated negatively with echocardiographic parameters of RV function such as tricuspid annular plane systolic excursion (TAPSE) and RV ejection fraction (Yang et al., 2014). In addition, CXCL10 and CXCL12 correlated significantly with RV end-diastolic diameter, mean right atrial pressure, and cardiac index (Yang et al., 2014). Recently, relationships between increased circulating IL-6 levels and RV function in PAH patients have been analyzed (Prins et al., 2017). Serum IL-6 levels correlated inversely with echocardiography-derived measures of RV function, including RV fractional area change, TAPSE and right ventricle–pulmonary artery coupling parameters (Prins et al., 2017). Importantly, these relationships remained significant, even after excluding patients with SSc-PAH (Prins et al., 2017). Remarkably, a negative relationship between circulating IL-6 and cardiac magnetic resonance imaging-derived RV ejection fraction was described (Prins et al., 2017). A significant relationship has also been demonstrated between circulating levels of TNF-α and severity of peripheral edema in patients with right heart failure related to ischemic heart disease or idiopathic dilated cardiomyopathy (Odeh et al., 2006). Further, a significant inverse correlation was found between serum TNF-α and multigated acquisition (MUGA) technique-derived RV ejection fraction (Odeh et al., 2006). In **Table 2**, we reported a list of inflammatory markers, which reflect adverse RV remodeling and dysfunction.

**Table 2:** Summary of studies evaluating the relationship of circulating inflammatory biomarkers with the parameters of right ventricular performance.

Disease	Sample	Population	Inflammatory mediators	Findings	References
CTEPH and CHF	Serum	Patients with CTEPH (*n* = 49), CHF (*n* = 17), Control (*n* = 34)	TNF-α, sTNFR-1 and -2, IL-10, hs-CRP, and NT-proBNP	High serum levels of TNF-α sTNFR1, sTNFR2, NT-proBNP, and IL-10 in CTEPH and CHF patients. Correlations between sTNFR1, sTNFR2, IL-6, hs-CRP, and NT-proBNP and magnetic resonance imaging-derived RVEF.	von Haehling et al., 2010
IPAH	Plasma	Patients with IPAH (*n* = 61), Control (*n* = 20)	CXCL-10, CXCL-12, and CXCL-16	Association of increased levels of CXCL-10, CXCL-12 and CXCL-16 with RVEF and TAPSE.	Yang et al., 2014
PAH	Serum	Patients with PAH (*n* = 40)	IL-6	Inverse correlation of serum IL-6 levels with echocardiography-derived RV FAC, TAPSE, and right ventricle–pulmonary artery coupling parameters. Negative relationship between circulating IL-6 and cardiac magnetic resonance imaging-derived RV ejection fraction.	Prins et al., 2017
HF patients presenting with RVF	Serum	HF patients with RVF (*n* = 83), Control (*n* = 15)	TNF-α	Correlation of TNF-α levels with severity of peripheral edema and multigated acquisition (MUGA) technique-derived RVEF.	Odeh et al., 2006

*PAH, pulmonary arterial hypertension; IPAH, idiopathic pulmonary arterial hypertension; CTEPH, chronic thromboembolic pulmonary arterial hypertension; CHF, chronic heart failure; RV, right ventricular; RVF, right ventricular failure; RVEF, right ventricular ejection fraction; RV FAC, right ventricular fractional area change; TAPSE, tricuspid annular plane systolic excursion; TNF-α, tumor necrosis factor alpha; sTNFR, soluble tumor necrosis factor receptors; IL, interleukin; CXCL, chemokine (C-X-C motif) ligand; NT-proBNP, oxN-terminal pro oxb-type natriuretic peptide; hs-CRP, high-sensitivity oxC-reactive protein*.

*RV, right ventricular; TNF-α, tumor necrosis factor alpha; sTNFR, soluble tumor necrosis factor receptors; NT-proBNP, oxN-terminal pro oxb-type natriuretic peptide; hs-CRP, high-sensitivity oxC-reactive protein; IL, interleukin; PTX3, pentraxin 3; CXCL, chemokine (C-X-C motif) ligand*.

## Targeting Inflammation

Low-level graded aerobic exercise is recommended as a general measure in the treatment of PH by the current European Society of Cardiology – European Respiratory Society Guidelines on Pulmonary Hypertension (Galie et al., 2016). There is substantial evidence for anti-inflammatory effects of physical activity in patients with various cardiovascular, metabolic, or pulmonary diseases (Pedersen, 2017). Beneficial effects of exercise training on pulmonary hemodynamics and functional capacity has also been shown in patients with PH (Arena et al., 2015; Buys et al., 2015; Babu et al., 2016; Ehlken et al., 2016). Moreover, acute effects of exercise on the inflammatory state in patients with idiopathic PAH have recently been reported (Harbaum et al., 2016). In a recent study using monocrotaline-induced PH in rats, high intensity interval training lowered RV systolic pressure, RV hypertrophy and fibrosis, and increased cardiac output (Brown et al., 2017). In another study, effects of continuous exercise training were found to be beneficial only in adaptive RV remodeling (Handoko et al., 2009). In contrast, in progressive PH with maladaptive RV remodeling, continuous exercise training worsened survival and dramatically increased RV leukocyte infiltration (Handoko et al., 2009). It is obvious that effects of exercise training depend on frequency, duration, and intensity of exercise. More studies are therefore necessary to explore a potentially more optimal exercise regimen and to investigate effects of training on RV adaptation/maladaptation. A randomized controlled trial to evaluate the effect of exercise training program on hemodynamics and cardiac magnetic resonance-derived parameters of RV function in patients with PAH (the ExPAH study) is currently underway (Chia et al., 2017).

Currently approved drugs used in the medical management of PAH target endothelin 1, nitric oxide, and prostacyclin pathways, which are important in the control of pulmonary vasomotor tone and vascular cell proliferation (Galie et al., 2016). Although none of the currently approved PAH-specific therapies primarily targets inflammatory mechanisms, there is evidence for anti-inflammatory properties of these drugs (Stasch et al., 2011; Stitham et al., 2011; Watzinger et al., 2016). There are several preclinical studies suggesting that PAH-targeted drugs might influence inflammatory processes in the pressure overloaded right ventricle. In dogs subjected to transient PAB, epoprostenol infusion limited activation of inflammatory processes in the right ventricle (Dewachter et al., 2015). Further, chronic treatment of rats with monocrotaline-induced PH with the endothelin-1 antagonist bosentan was associated with improved RV function and preserved right ventricle-pulmonary artery coupling and reduced cytokine levels in the right ventricle (Fontoura et al., 2014).

Recent experimental studies have demonstrated efficacy of direct anti-inflammatory drugs, including TNF-α antagonists (Wang et al., 2013), anti-CD20 antibodies (Mizuno et al., 2012; Breitling et al., 2017), an inhibitor of T helper 17 cell development SR1001 (Maston et al., 2017), and mast cell stabilizers (Dahal et al., 2011; Bartelds et al., 2012), in attenuating PH. Phase II clinical trials investigating novel drugs targeting inflammation and immune dysfunction in PAH (ubenimex, rituximab, and tocilizumab) are currently underway (Lau et al., 2017). However, data on the effects of anti-inflammatory agents on adverse RV remodeling and dysfunction are scarce. In experimental acute RV failure following PE, several studies have shown that various anti-inflammatory strategies, including treatment with antibodies against polymorphonuclear leukocytes, antibodies to CXCL-1, or anti-inflammatory drug ketorolac can decrease expression of RV inflammatory genes, reduce neutrophil influx, and prevent RV dysfunction (Watts et al., 2006, 2009; Zagorski et al., 2007). Similarly, in rats with chronic RV pressure overload following PAB, treatment with the NF-κB inhibitor pyrrolidine dithiocarbamate decreased macrophage accumulation in the right ventricle, which was associated with improved RV remodeling and function (Yoshida et al., 2016).

## Concluding Remarks

**FIGURE 1:**
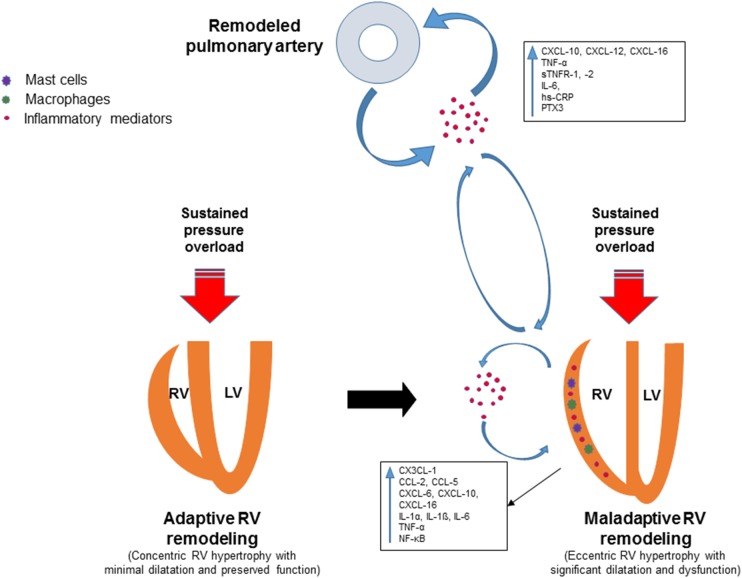
Schematic view of the involvement of inflammatory mediators in adverse right ventricular remodeling and dysfunction. Remodeling of pulmonary vessels in PAH is induced by various pathological mechanisms including inflammatory and autoimmune processes. Circulating inflammatory mediators originating from the pulmonary vasculature may trigger or contribute to inflammatory processes in the pressure-overloaded right ventricle. Inflammation in the right ventricle is characterized by upregulation of pro-inflammatory cytokines and chemokines, expansion of leukocytes, macrophages and mast cells. Upregulated inflammatory mediators contribute to adverse right ventricular remodeling and dysfunction through various mechanisms.

A plethora of inflammatory mediators are upregulated in the right ventricle in response to pressure overload along with accumulation of inflammatory cells (**Figure 1**). However, the exact role of the inflammatory mediators in this context is not established yet. Further studies are required to gain mechanistic insight into how these mediators contribute to maladaptive RV remodeling and dysfunction. A better understanding of the role of inflammation in the pathogenesis of adverse RV remodeling and failure may lead to novel anti-inflammatory therapies for selected patients. Biomarker strategies identifying patient subpopulations with overactive pro-inflammatory signaling may contribute to rational implementation of anti-inflammatory therapies to prevent or reverse RV maladaptive remodeling and dysfunction. Preliminary evidence from preclinical studies suggests that therapeutic approaches targeting specific components of the inflammatory response may be promising for patients with pressure overload-induced adverse RV remodeling and dysfunction.

## Author Contributions

AS, AM, AP, and DK drafted the manuscript. AS, AM, AP, DK, ASS, HG, NW, and RS revised the manuscript critically for important intellectual content. AS, AM, AP, DK, ASS, HG, NW, and RS approved the final version of the manuscript submitted.

## Conflict of Interest Statement

The authors declare that the research was conducted in the absence of any commercial or financial relationships that could be construed as a potential conflict of interest.
